# Computer‐aided Design of Distal Femoral Osteotomy for the Valgus Knee and Effect of Correction Angle on Joint Loading by Finite Element Analysis

**DOI:** 10.1111/os.13440

**Published:** 2022-09-24

**Authors:** Yanfei Wu, Xin Jin, Xingwen Zhao, Ying Wang, Haohao Bai, Bin Lu, Xue Tong, Jianxiong Ma, Xinlong Ma

**Affiliations:** ^1^ Clinical College of Orthopedics Tianjin Medical University Tianjin China; ^2^ Tianjin Hospital, Tianjin University Tianjin China

**Keywords:** Cartilage, Finite element analysis, Meniscus, Shear stress, Von Mises

## Abstract

**Objective:**

Lateral open‐wedge distal femoral osteotomy (DFO) has been used to treat valgus deformity of the knee, with good clinical outcomes. However, there is a lack of biomechanical studies regarding the angle of correction. The objective of this study was to apply computer‐aided design (CAD) for osteotomy planning in a three‐dimensional (3D) anatomical model and to assess the biomechanical differences among the varying correction angles on joint loading by finite element analysis (FEA).

**Methods:**

To model different angles of lateral open‐wedge DFO correction, the CAD software package Mimics 21.0 was used to accurately simulate the operated knee. The femur was cut to 0°, 2°, 4°, 6°, 8°, and 10° of varus (equivalent to hip‐knee‐ankle angles of 180°, 178°, 176°, 174°, 172°, and 170°, respectively). The original knee model and the corrected models were processed by FE software. Then, the FE models were subjected to an axial force to obtain the von Mises stress (VMS) and shear stress distributions within the femoral cartilages and menisci.

**Results:**

Under a compressive load of 740 N, the highest VMS in lateral and medial compartments of the intact knee model was 3.418 and 3.303 MPa. The maximum value of both the VMS and the shear stress in the lateral compartment decreased as the varus angle increased, but the corresponding values in the medial compartment increased. When the hip‐knee‐ankle (HKA) angle was 180°, the VMS in the lateral and medial compartments was balanced (3.418 and 3.303 MPa, respectively). Meanwhile, when the HKA angle was 178° (3.488 and 3.625 MPa, respectively), the shear stress in the lateral and medial compartments was balanced. In addition, the magnitude of change in the stress was significantly higher in the medial compartment (90.9%) than in the lateral compartment (19.3%).

**Conclusion:**

The optimal correction angle of the valgus knee is close to neutral alignment or slightly varus (0° ‐ 2°). Overcorrection is not recommended, as it can result in a steep increase of the stress within the medial compartment and may accelerate the process of medial compartment OA.

## Graphical abstract

This study combined computer‐aided design (CAD) with finite element analysis (FEA) to investigate the effect of simulated lateral open‐wedge distal femoral osteotomy (DFO) with varying correction angles on stress distributions within the cartilages and meniscus. This individualized non‐invasive analysis of stress distribution provided a promising novel approach for clinical evaluation of the mechanical loading of cartilage and meniscus.

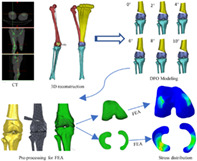

## Introduction

Valgus deformity of the knee is less commonly encountered but more technically challenging than varus deformity.^1^ Distal femoral osteotomy (DFO) is predominantly used for mild to moderate valgus deformity, with positive short‐term and long‐term clinical outcomes, and even reduces the need for joint replacement.^2^, ^3^ It has also been demonstrated that restoration of the biomechanical axis reduces the levels of inflammatory factors within the knee joint, which could provide a favorable environment for cartilage regeneration.^4^ Valgus deformity of the knee is a complex of 3D deformities that require adequate correction in all three dimensions, on the coronal, sagittal, and axial planes. However, the accuracy of traditional osteotomy planning with plain X‐ray radiography is unsatisfactory. Suppose the patient is not placed in a standard position. In that case, the femur may be internally or externally rotated. The femoral head may not show a complete circle, leading to inaccurate central positioning and errors in the preoperative mechanical axis planning, thus affecting the effect of surgical correction. In addition, a two‐dimensional (2D) anteroposterior radiograph tends to ignore deformities in the sagittal and axial positions.^5^ Therefore, we need a new approach to improve these problems. With the rapid advances in image‐based computer‐aided technology, CAD is widely applied in diagnosis or preoperative surgical planning, allowing for more precise establishment of the mechanical axis.^6^ In addition to accurate measurement, an appropriate correction angle is also crucial to clinical outcomes.^7^ However, the current literature lacks a consensus concerning the optimal correction angle based on knee biomechanics. The prevailing view is that^8^, ^9^ when the lower‐limb alignment is close to neutral, the balance of stress between the lateral and medial compartments of the knee is close to physiological. However, Quirno *et al*.^10^ have suggested that surgeons should perform the osteotomy with 5° of overcorrection to restore near‐normal contact pressures in the lateral compartment.

To date, there have been many achievements in the study of the normal knee joint based on the FE method,^11^, ^12^, ^13^ but pathological knee joint models have rarely been reported. Hence, the main intention of the present study was: (i) to construct a 3D simulation model of valgus deformity and DFO models of varying correction angles with CAD; and (ii) to investigate the stress distributions within the knee compartments and compare the impact of different DFO correction angles on the stress distributions within the knee compartments with FEA.

## Materials and Methods

### Imaging Data Acquisition

**Fig. 1:**
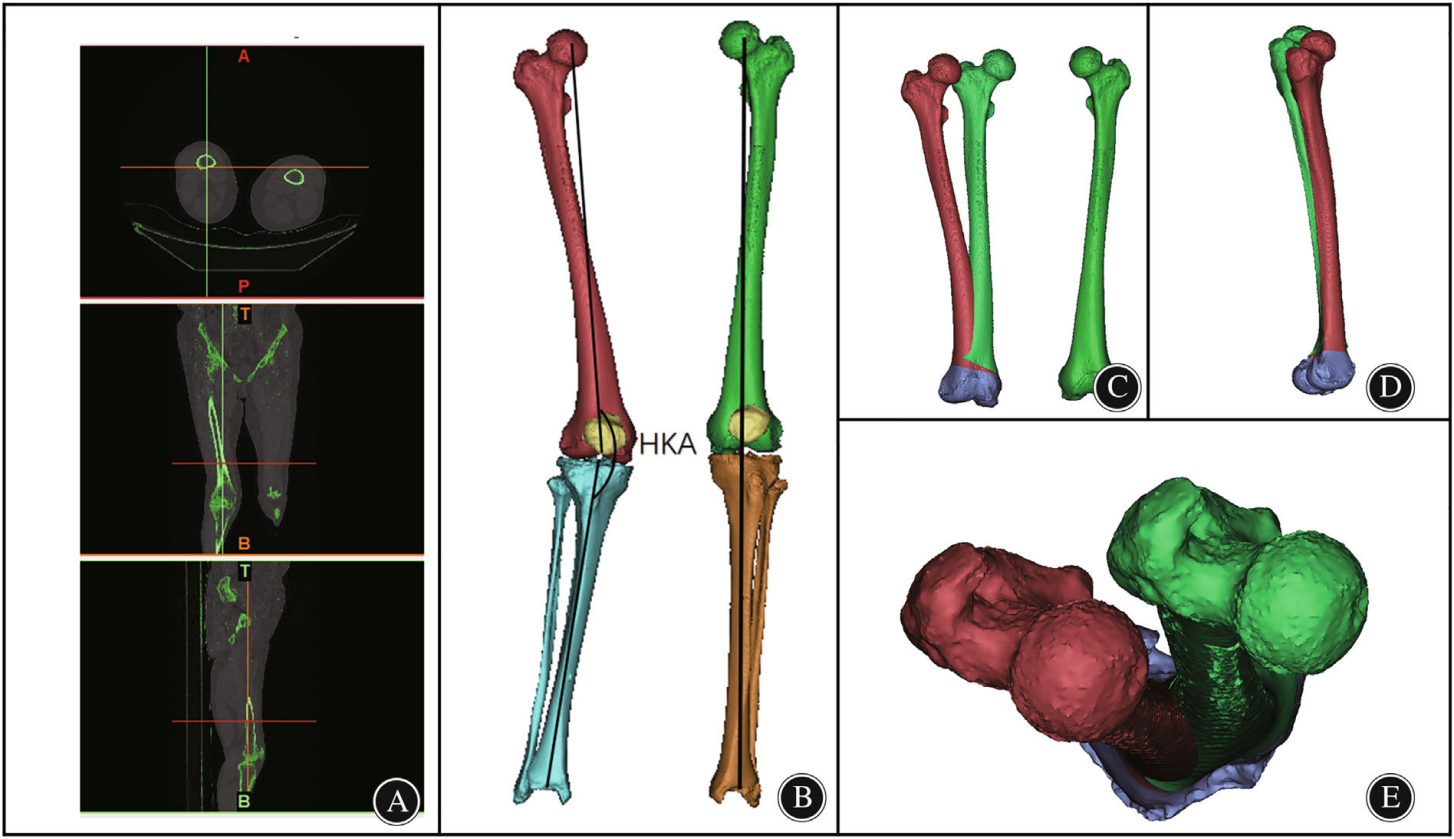
3D CT reconstruction. (A). CT images of both lower limbs. (B). 3D skeletal reconstruction of the lower limbs and mechanical axis of the lower limbs. (C). Coronal view of the reconstructed right femur (red), mirrored left femur and left femur(green). (D). Sagittal view of the right femur (red) and mirrored left femur(green). (E). Axial view of the right femur (red) and mirrored left femur(green).

One volunteer with valgus deformity and mild OA in the right knee (female, 55 years old, 70 kg) underwent computed tomography (CT) and magnetic resonance imaging (MRI) of the knee joint with the leg in an extended position, a CT (Lightspeed VCT; GE, Las Vegas, NV, USA) was performed with a layer thickness is 0.625 mm, and MRI (SIGNA 3.0T; GE, Las Vegas, NV, USA) was performed with a layer thickness of 3 mm using cross‐sectional T2‐weighted imaging (T2WI) and sagittal T1‐weighted imaging (T1WI) sequences The CT and MRI data of the patient were imported into Mimics 21.0. Based on CT images with different thresholds, the bone was segmented. The masks of the femur, tibia, fibula and patella were segmented by using the region‐growing function, and the corresponding 3D bone models were generated (Fig. 1A,B).

### Preoperative Measurements Based on the 3D Model

The femoral mechanical axis was defined as the line from the center of the femoral head through the center of the knee, and the mechanical axis of the tibia was defined as the line from the center of the ankle through the center of the knee (Fig. 1B).^14^ The HKA angle was defined as the medial angle between the mechanical axis of the tibia and the femur,^15^ which represents lower‐limb alignment. Neutral alignment of the knee is represented by an HKA angle of 180°, while varus and valgus knee deformity is generally represented by an HKA angle <180° and >180°, respectively. The mechanical axes of the left and right lower limbs were drawn. As shown in Fig. 1B, the right lower limb showed obvious knee valgus (HKA angle = 194°), and the left lower limb showed no obvious abnormality (Fig. 1B). Thus the left lower‐limb alignment was used as the standard of reference for correcting the deformity of the right lower limb. The left femur was mirrored to create an ideal right femur using the mirroring function of Mimics software. After the 3D visualization process, obvious coronal deformity and mild sagittal deformity were found (Fig. 1C,D), while no obvious axial deformity was found (Fig. 1E).

### DFO Modeling

**Fig. 2:**
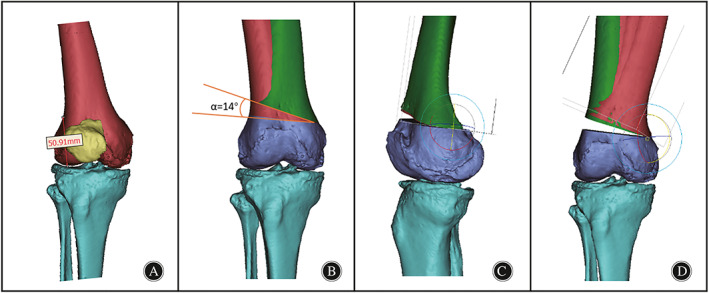
DFO model construction. (A). Placement of the osteotomy point was taken at the superior border of the patella (50.9 mm from the distal articular surface). (B). Right femur with a 14° angle from the ideal femur on the coronal plane. (C). Deformity correction of the knee on the sagittal plane; (D). Rotation of the femur on the coronal plane to establish the corrected and overcorrected models.

Under the guidance of experienced orthopedic surgeons and based on preoperative measurements, a lateral open‐wedge DFO was performed. The osteotomy plane is generally located 5–6 cm above the femoral articular surface (equivalent to the plane of the upper edge of the patella) (Fig. 2A). The osteotomy began in the lateral supracondylar area and ended in the medial femoral condyle (Fig. 2B). The right femur and the ideal right femur were superimposed on each other using Mimics software. Then, the right femur was divided along the osteotomy plane and rotated on the sagittal plane until it was closely aligned with the ideal right femur (Fig. 2C). Finally, for investigative purposes, we continued to rotate the femur model on the coronal plane to establish 3D models of neutral (equivalent to 0° varus) and varus (2°, 4°, 6°, 8°, and 10°) positions.

### FE Model Establishment

**Fig. 3:**
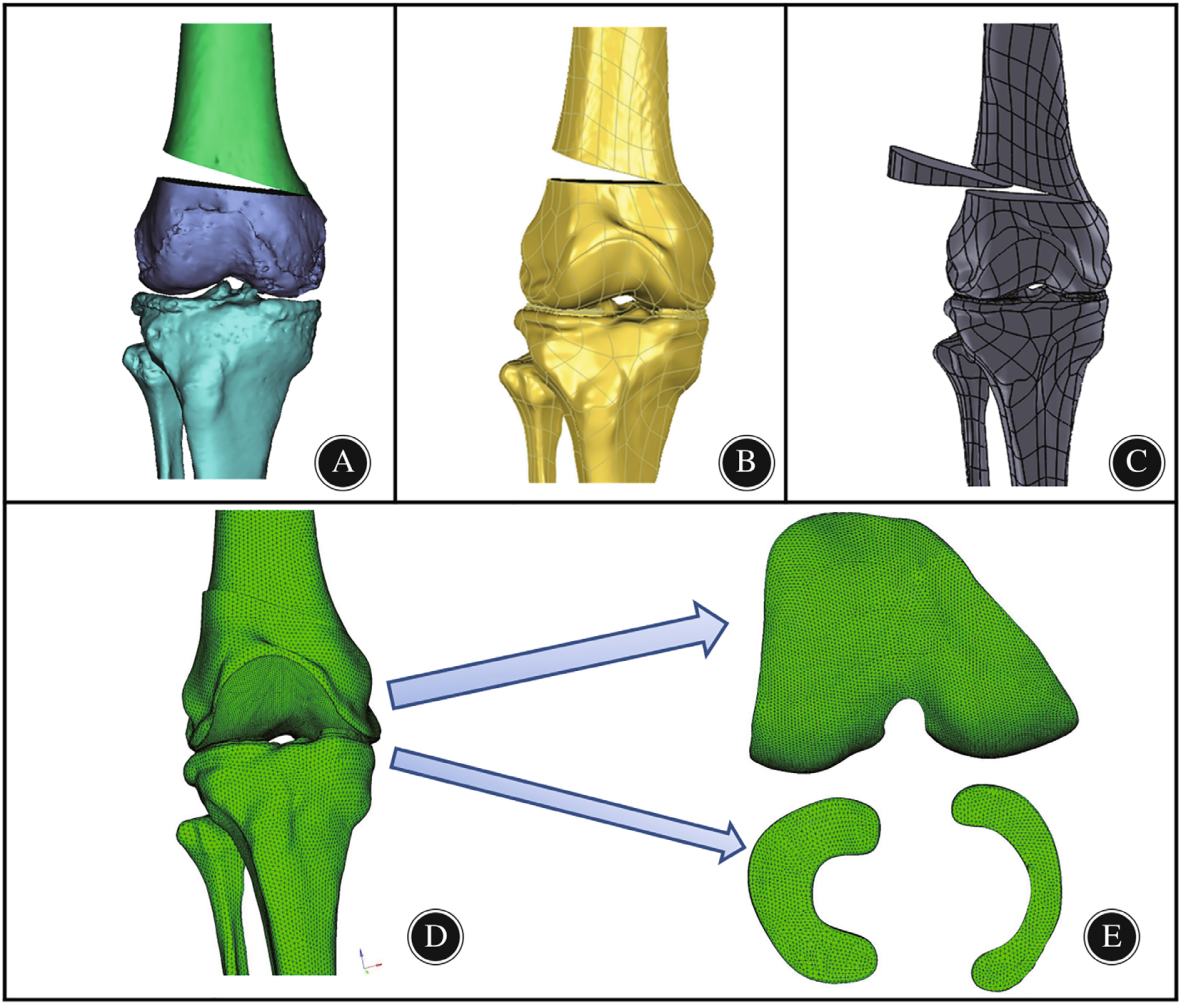
Finite element pre‐processing step for the neutrally aligned knee. (A). the osteotomy model was constructed with Mimics software; (B). the reconstructed model was smoothed and converted to CAD models in Geomagic studio software; (C).bone graft was designed and assembled via SolidWorks; (D). the intact knee model was meshed in HyperMesh software using 4‐node tetrahedron elements. (E). the subdivision of the mesh of cartilage and meniscus.

To simplify the model and facilitate FEA calculations, we made the assumptions that the period of bone healing after DFO has been completed and that screws and plates have been removed (Fig. 3A). The 3D structure of the femoral cartilage, ligaments, and other tissues is complex. By repeatedly comparing them on multiple views, we manually separated the masks of soft tissues including femoral cartilage, tibial cartilage, and menisci, on both sides, generated the corresponding 3D model, performed CT/MRI image coregistration, fused the 3D model of bony and nonbony structures, and assembled them in the same spatial coordinate system according to their anatomical positions in vivo. The models of varying correction angles underwent identical manipulation. Then, the models were exported to STL file format followed by conversion with smooth regular triangular surface structures without holes or triangles, to IGES format using Geomagic studio (Geomagic Solutions, Cary, NC, USA) (Fig. 3B). The models processed by Geomagic were imported into SOLIDWORKS to design the corresponding bone graft (Fig. 3C). All models with IGES format files were imported into HyperMesh13.0 (Altair, Frisco, TX, USA), which was used to produce the mesh basis for the FE model. The average mesh size of bone was set to 2 mm, while that of cartilage and menisci was set to 1 mm(Fig. 3E).

### Material Assignment and Contact Setting

The meshed models were then exported as INP files and imported into an Abaqus 6.14 (SIMULIA, Paris, France) to calculate the stress distributions. In our work, the femur, fibula, and tibia were defined as isotropic linearly elastic materials. The cartilage and menisci were also defined as linear elastic and isotropic materials. The mechanical properties of the bone, menisci, and cartilage surrounding the bone are presented in Table 1. The ligaments were defined as linear spring elements connecting the femur to the fibula and tibia. Each ligament was composed of two springs with rigidity of 800 N/mm per spring.^18^

**TABLE 1:** Material properties of components in finite element model

Components	Young's modulus (MPa)	Poisson ratio
Bone^16^	20,000	0.3
Cartilage^16^	5	0.46
Meniscus^17^	59	0.49

### Loads and Boundary Conditions

The distal ends of the fibula and tibia were fixed in all degrees of freedom, while the femur was free in all degrees of freedom. The contacts were modeled between the femoral cartilage and meniscus, and between the femoral cartilage and tibia cartilage for both the medial and lateral sides, resulting in four contact pairs.^19^ The interface between the femoral and tibia cartilage and between the femoral cartilage and menisci was considered to exhibit frictionless surface‐to‐surface contact, including finite sliding. The contact pressure–clearance relation for defining interactions between the surfaces was set as “hard” contact. The other contacts were applied as tied constraints to simulate the junction of the knee joint. To facilitate comparison with existing literature, a uniaxial force of approximately 740 N was imposed at the coupling point of the proximal femur, which was equivalent to the weight of the female. Finally, the VMSs of menisci and cartilage under different correction angles were calculated by a single axial force. All models were assigned the same loading and boundary conditions.

### Evaluating Parameters

The peak VMS distribution in the menisci and femoral cartilage was recorded and considered to indicate the risk of cartilage damage and meniscal tears. Shear stress generated by compression harms cartilage matrix production and decreases biosynthetic activity of cartilage, resulting in cartilage damage.^20^ Thus, shear stress (Tresca equivalent stress) in this study was applied to assess the susceptibility to cartilage degeneration. Furthermore, 10 nodes were selected at 3 mm from the center of each cartilage contact surface, and the VMS of nodes was counted to evaluate the change in stress values further.

#### Statistical Analysis

Statistical analysis comparing the intact model and osteotomy models was performed using a paired Student's *t*‐test or non‐parametric test, calculated with SPSS 25.0 software (SPSS Inc., Chicago, IL, USA). Statistical significance was designated as *P* < 0.05.

## Results

### Validation of the Knee Model

Because of the scarce number of reported studies about valgus knee models, we could only compare the VMS of the neutrally aligned knee model with those intact knee models reported in the literature. In our study, under a compressive load of 740 N, the highest VMS in lateral and medial compartments of the intact knee model was 3.418 and 3.303 MPa, respectively.

### Impact of the Correction Angle on the VMS Distribution

**Fig. 4:**
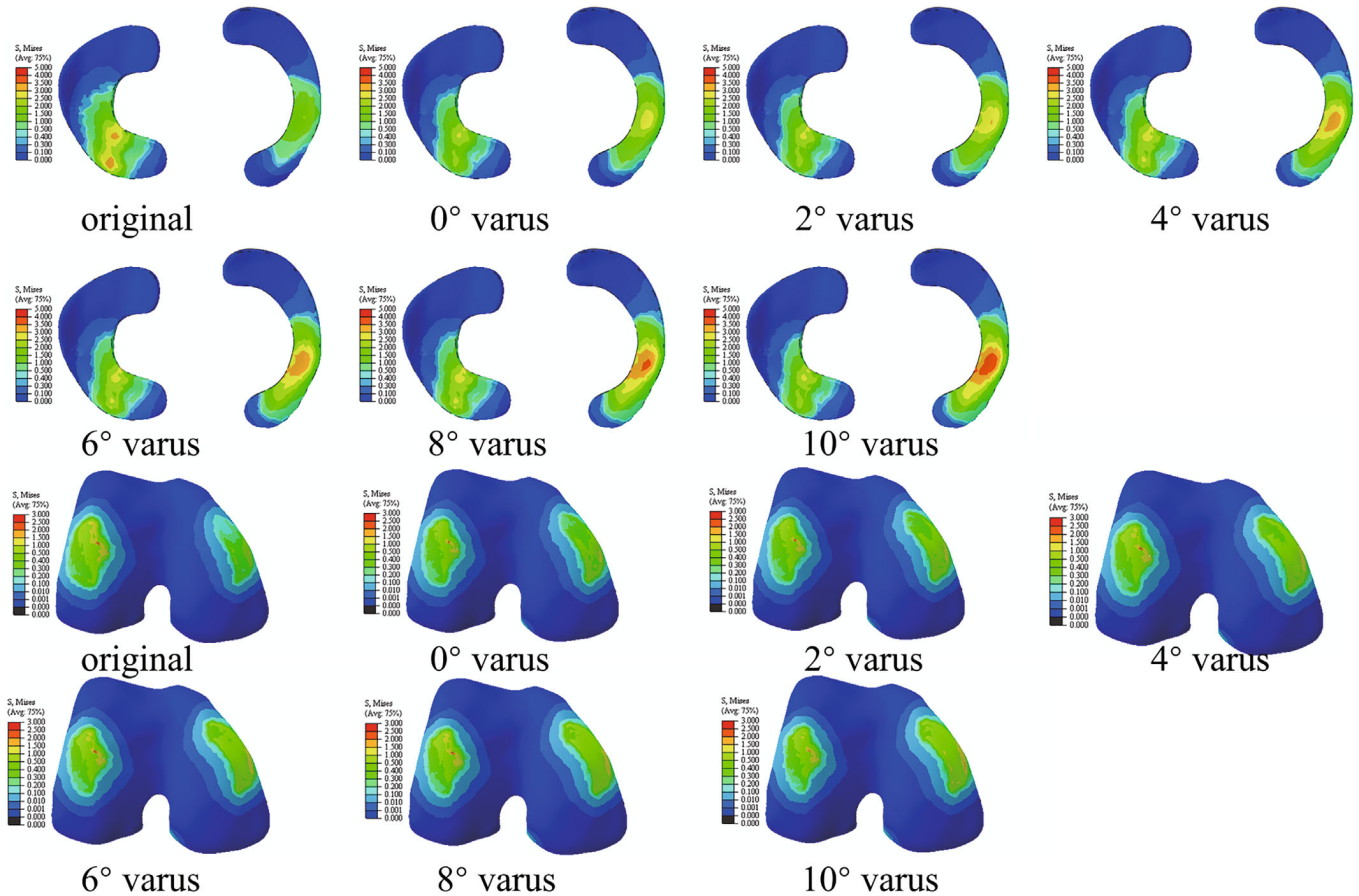
The peak VMS stress distribution of meniscus and femoral cartilage of all models. Colors represent stress values from minimal (blue) to maximal (red).

**Fig. 5:**
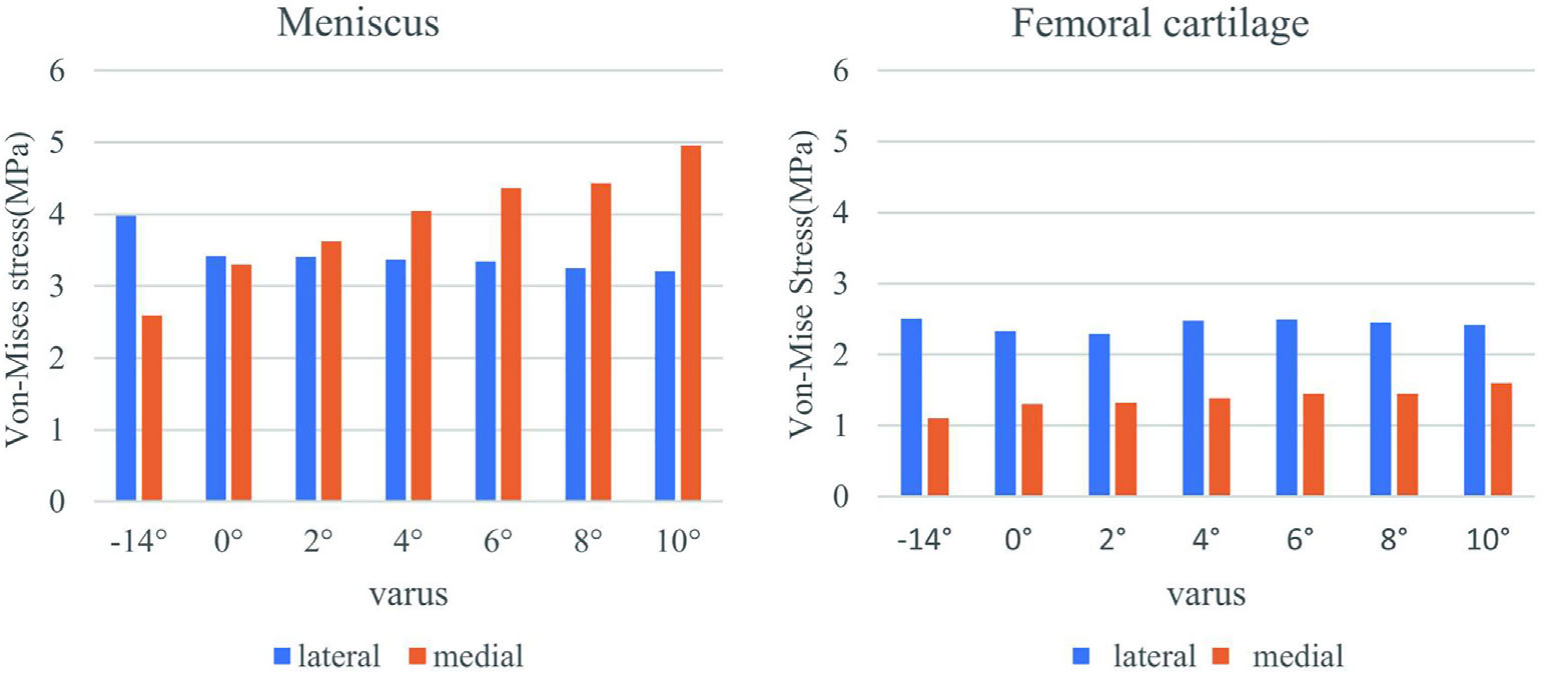
Variation of the peak VMS in the meniscus and femoral cartilage of lateral compartment and medial compartment as the increase of varus correction angle.

**Fig. 6:**
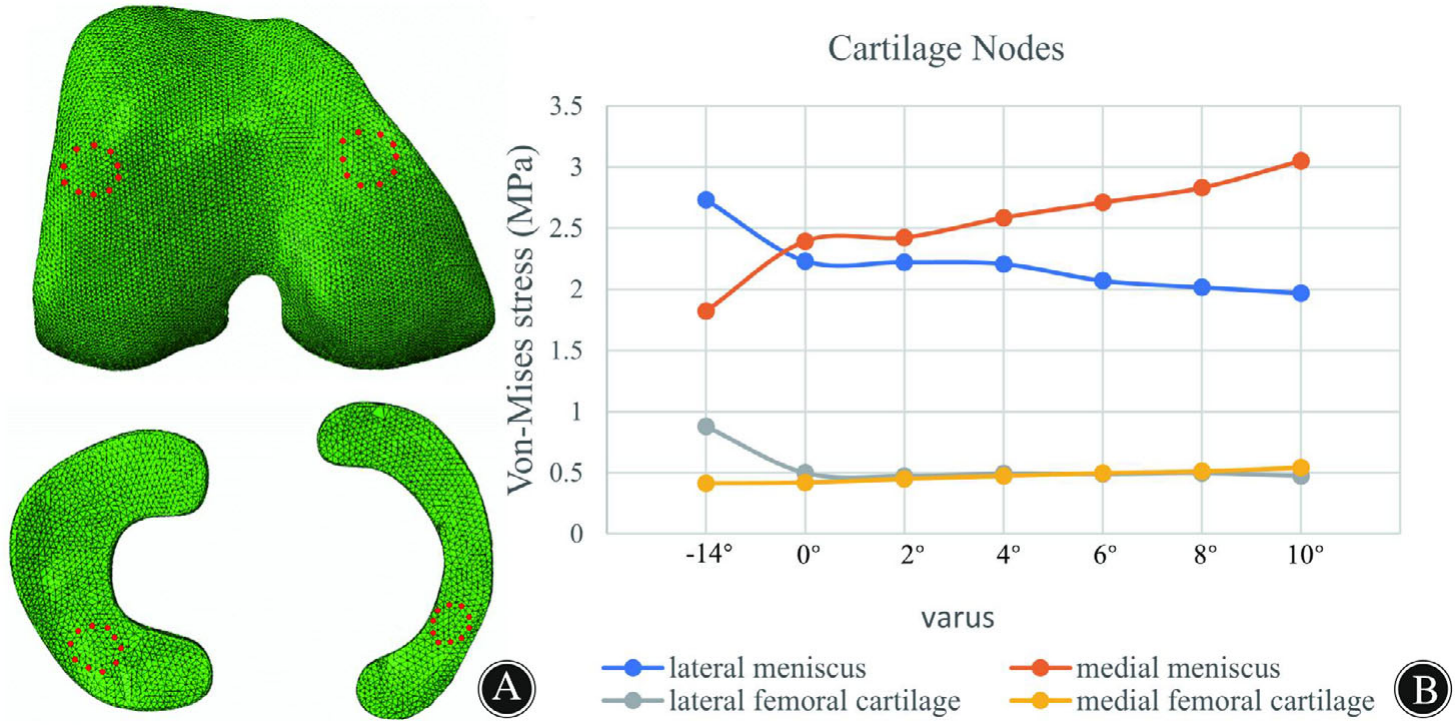
(A) Ten nodes of the meniscus and femoral cartilages; (B) Changes in mean VMS of cartilage nodes in different corrected models.

The maximum stress in the lateral and medial compartments was located in the anterior half of the lateral meniscus and medial meniscus, respectively (Fig. 4). The peak VMS in the lateral meniscus decreased by 19.3% from 3.977 to 3.208 MPa, while that in the medial meniscus increased by 90.9% increase from 2.595 to 4.954 MPa when the correction angle changed from −14° (that is, 14° valgus) to 10° varus (Fig. 5). The VMS in the medial femoral cartilage increased from 1.102 to 1.601, while that in the lateral femoral cartilage varied irregularity over the range of correction. A substantially balanced stress distribution between two compartments was attained when the HKA was corrected to 0° varus (Fig. 5). The average VMS values of the 10 nodes of the meniscus and femoral cartilage in different osteotomy models were compared and shown in Fig. 6. We found that as the varus angle increased, the average VMS values of the lateral meniscus and lateral femoral cartilage tended to decrease, while that in the medial meniscus and medial femoral cartilage tended to increase. The results showed statistically significant difference between all osteotomy models (0–10° varus) compared to the intact model (−14° varus) (*P* < 0.05).

### Impact of the Correction Angle on the Shear Stress Distribution

**Fig. 7:**
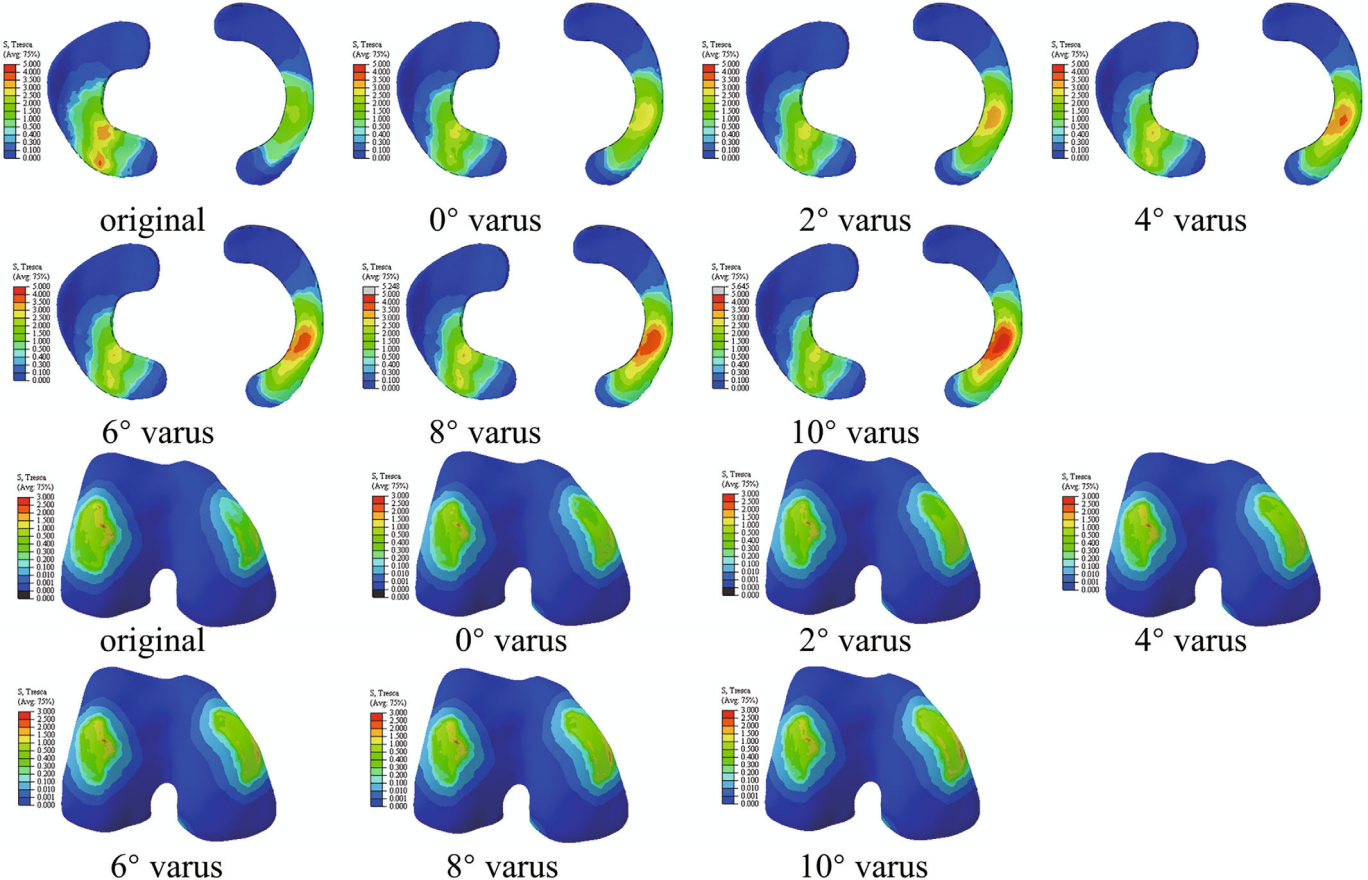
The peak shear stress distribution of meniscus and femoral cartilage of all models.

**Fig. 8:**
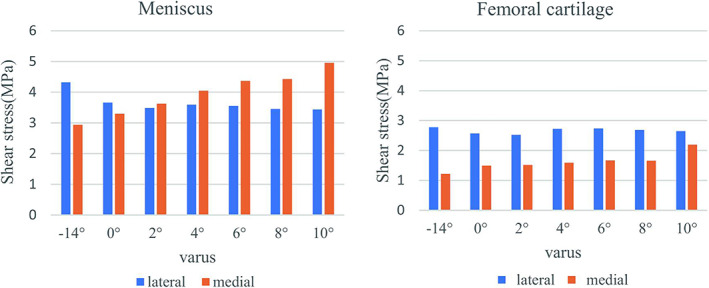
Variation of the peak shear stress in the meniscus and femoral cartilage of lateral compartment and medial compartment as the increase of varus correction angle.

The peak shear stress in the medial and lateral compartments was also concentrated on the menisci (Fig. 7). When the correction degree varied from −14 to 10° varus, the peak shear stress of the lateral meniscus decreased from 4.326 to 3.442 MPa, a decrease of 20% (Fig. 8). Meanwhile, the shear stress of the medial meniscus was observed to increase from 2.939 to 4.954 MPa, an increase of 68.6%. The stress of the medial femoral cartilage gradually increased from 1.222 to 2.191 MPa. The stress of lateral femoral cartilage showed a trend of falling first and rising again, with *the* maximum value at −14° varus (2.776 MPa) and the minimum value at 2° varus (2.521 MPa). In addition, when the HKA angle was 178° (2° varus), a substantially balanced shear stress distribution between the lateral and medial compartments was attained.

## Discussion

This study combined CAD with FEA to investigate the effect of lateral open‐wedge DFO with different correction angles on stress distributions within the femoral cartilage and menisci. We found that the maximum and mean values of VMS in the lateral compartment showed an increasing trend as the varus angle increased, while that in the medial compartment showed an increasing trend. Furthermore, the stress change magnitude was significantly higher in the medial compartment than in the lateral compartment.

### Advantage of DFO Modeling based on CAD

The main novelty of this study was simulating DFO with CAD software to establish 3D correction models, which are more precise than the conventional approach of correction on the frontal plane.^21^ The traditional osteotomy plan is based on the anatomical information available from 2D radiographs. For patients with complex deformities, it is difficult to achieve a satisfactory outcome using the traditional preoperative osteotomy plane; thus, computer‐aided 3D reconstruction could be considered for preoperative planning of the osteotomy scheme. The CAD software Mimics is capable of various manipulations of 3D images, such as scaling, free locomotion, and rotation, which could facilitate the more intuitive design of osteotomy plans. Thus, CAD techniques could provide accurate FE osteotomy models for investigating the effect of the correction angle on articular loading.

The goal of DFO is to restore the balance of loading between two compartments of the knee.^22^, ^23^ Performing DFO for correcting valgus deformity is a good surgical option for severe cases. ^2^, ^24^, ^25^ Shivji *et al*.^26^ reported that 86 patients with an average age of 48 years were treated with DFO due to valgus knee deformity combined with lateral‐compartment OA. The 10 year joint survival rate was 89%. DFO has yielded successful results in the treatment of lateral‐compartment OA in clinical studies, but few studies have investigated the biomechanical effects of DFO. A literature search produced only one article, by Quirno *et al*.,^10^ describing the biomechanical effects of DFO. The results of mechanical experiments on cadaver specimens have indicated that 5° of correction is better for restoring contact areas and contact pressures in the lateral compartment. However, the biological properties of many tissues of cadavers are altered due to immersion in chemicals, exposure to drugs, and detachment of accessory tissues, decreasing the accuracy of the results. In vivo testing is limited by the number and quality of specimens. The FE method is a method for establishing models through computer software, which can be used to simulate the mechanical characteristics of the knee.^27^

### Validity and Significance of FE Models

The highlight of the present study is that the FE models are based on a real clinical case rather than remodeling a normal case in the literature.^11^, ^21^ In a study by Trad *et al*.,^21^ the highest VMS in the lateral and medial compartments was 1.93 and 2.65 MPa, respectively, under a compressive load of 740 N. In a study by Zheng *et al*.,^28^under a compressive load of 740 N, the highest stress in the knee compartments was 3.06 MPa. In comparison with data from our study, the main reason for the minor discrepancy may be individual differences in model origin, such as the thickness of cartilage and menisci in each study.^29^ However, in general, the results of our study and previous studies in the literature are in reasonable agreement, indicating that the results obtained by analysis of the FE model constructed in the present study are reliable. More significantly, the individualized noninvasive and quantitative method for stress distribution analysis offers a promising novel approach for assessing cartilage loading. As shown in Figss 4 and 6, the stress concentrations are generally near areas of meniscal wear, which is consistent with clinical observations. The concentration of loading stress increases cartilage wear, creating a vicious cycle that accelerates the development and severity of OA. Clinical and biomechanical studies have supported that restoration of the mechanical axis can effectively relieve the development of OA,^30^ which is why the lateral open‐wedge DFO was considered in this study.

### Investigation of Stress Distributions within the Knee Compartments

In this study, we applied FEA to quantify alterations in the biomechanics of the knee. The peak VMS was applied to quantitatively assess the severity of wear of the menisci and cartilage. Additionally, according to the literature, peak shear stress is considered to play a major role in accelerating cartilage degeneration.^31^ Our study demonstrates that DFO correction from −14 to 10° varus apparently reduced stresses in the lateral compartment and increased stresses in the medial compartment. Moreover, the recommended HKA angle was found to be 180° when considering VMS and 178° when considering shear stress. However, there have been no studies investigating which stress should be the standard for deciding the correction angle. According to the von Mises criterion, the VMS is the combined effect of tensile stress, compressive stress, and shear stress.^32^, ^33^ Compressive stress closely associated with pain has been widely recognized as a greater factor in correctional osteotomy. Therefore, the VMS may be a more useful guide for surgeons to determine the correction angle.

Another key finding of the study indicated a greater magnitude of change in stress in the medial compartment (i.e., 90.9% for VMS, 68.6% for shear stress) than in the lateral compartment (i.e., 19.3% for VMS, 20% for shear stress) as the varus angle increased, which may be related to an increase in the knee adduction moment. This result also suggested that overcorrection would increase the possibility of medial OA.^34^ However, the stress of the lateral femoral cartilage did not gradually decrease but reached a minimum at 2° varus, which was unexpected and may be related to the change in tension of the spring unit used to simulate ligament tension. The long‐term valgus state places the lateral ligament in a relatively contracted state, and during the valgus correction, the tension of the lateral ligament gradually increases, while the load on the lateral compartment gradually decreases due to the inward shift of the mechanical axis; thus, overall, the pressure on the lateral compartment still tends to decrease. When corrected to 2° varus, the lateral collateral ligament is hypertonic, while the medial collateral ligament is relatively lax, resulting in a mild tendency toward increased lateral stress.

### Limitations

Some limitations of this work must be recognized. This study was carried out by only considering a uniaxial load. Therefore, we did not consider the dynamic mechanical behavior of the models. Moreover, simplifications were made regarding the knee model and details of DFO and the impacts of surrounding soft tissues were ignored. Therefore, the menisci and cartilage may be subjected to additional stress if the actions of the surrounding muscles and ligaments are considered in the knee model.^35^ The selection of a uniform bone density and uniform material properties (linear elastic, homogeneous, isotropic) for all of the knee components is another limitation of this study. As long‐term effects were the preferred focus of our work, immediate ligament tension changes were not included. Indeed, the application of a hyperelastic property of the ligaments and the nonlinear property of the menisci in the knee model would contribute to more precise results,^36^, ^37^ which would require further investigation in the future.

#### Conclusion

By comparing stress distributions within knee models with different degrees of correction, this study demonstrates that the optimal correction angle of the valgus knee is close to neutral alignment or slightly varus (0° ~ 2°). Overcorrection is not recommended, as it can result in a steep increase of the stress within the medial compartment and may accelerate the process of medial compartment OA. Furthermore, more patients need to be studied to determine if similar stress distributions exist in order to draw more precise biomechanical conclusions. Additional clinical imaging studies or cadaveric biomechanical investigations are warranted to verify these findings. Although the generality of our findings is limited, we provide a method that is expected to be a valuable tool in the preoperative planning of DFO surgery for valgus knee deformity.

## Author Contributions

Yanfei Wu, Jianxiong Ma, and Xinlong Ma contributed in the conception and design of study. Yanfei Wu and Xingwen Zhao contributed in data acquisition. Ying Wang, Bin Lu, Haohao Bai, and Xin Jin contributed to the execution of the experiments. Yanfei Wu and Xingwen Zhao analyzed and interpreted the data and drafted the manuscript. All the authors approved the final version of the manuscript to be published. All authors listed meet the authorship criteria according to the latest guidelines of the International Committee of Medical Journal Editors. All authors are in agreement with the manuscript.

## Funding Information

This work was supported in part by the National Natural Science Foundation of China (grant Nos. 11772226, 81871777, and 81572154), and in part by Tianjin Science and Technology Program (grant Nos. 18PTLCSY00070 and 16ZXZNGX00130).

## Conflict of Interest

There were no conflicts of interest in this study.
